# Treatment and Companion Diagnostics of Lower Back Pain Using Self-Controlled Energo-Neuroadaptive Regulator (SCENAR) and Passive Microwave Radiometry (MWR)

**DOI:** 10.3390/diagnostics12051220

**Published:** 2022-05-12

**Authors:** Alexander Viktorovich Tarakanov, Alexander Alexandrovich Tarakanov, Tatyana Kharybina, Igor Goryanin

**Affiliations:** 1 Department of Emergency Medicine, Rostov State Medical University, Rostov-on-Don 344022, Russia; dr-tarakanov@yandex.ru (A.V.T.); scenar.neuro@gmail.com (A.A.T.); 2 Library for Natural Sciences of the Russian Academy of Sciences, Moscow 119991, Russia; natsl@vega.protres.ru; 3 Biological Systems Unit, Okinawa Institute Science and Technology, Okinawa 904-0495, Japan; 4 School of Informatics, University of Edinburgh, Edinburgh EH8 9JS, UK

**Keywords:** lower back pain, passive microwave radiometry (MWR), transcutaneous electrical neurostimulation (TENS), self-controlled energy-neuro-adaptive regulation

## Abstract

Evaluation of the effectiveness of treatment of nonspecific lower back pain (LBP) is currently largely based on the patient’s subjective feelings. The purpose of this study was to use passive microwave radiometry (MWR) as a tool for assessing the effectiveness of various treatment methods in patients with acute and subacute nonspecific LBP. Patients with a pain assessment on a visual analogue scale (VAS) of 6 to 10 points were divided into two groups: Group I included patients with pharmacological, syndrome-oriented treatment (n = 30, age 54.9 ± 2.3 years); Group II included a combination of pharmacotherapy with self-controlled energy-neuroadaptive regulation (SCENAR) (n = 25, age 52.8 ± 2.5 years). The analysis showed that the addition of SCENAR therapy (Group II) significantly potentiated the analgesic effect at the stages of treatment, and after 3 weeks, this had increased by more than two times, by 1.3 points on the VAS. There was also a significant decrease in the maximum internal temperature and normalization of the gradient of internal and skin temperatures, and a decrease in thermo-asymmetry, as assessed by temperature fields. Thermal asymmetry visualization allows the identification of the area of pathological muscle spasm and/or inflammation in the projection of the vertebral-motor segment for the possible targeted use of treatment methods such as percutaneous electro neurostimulation, massage, manual therapy, diagnostic and treatment blocks, etc. The MWR method also avoids unnecessary radiation exposure.

## 1. Introduction

According to the Global Burden of Disease Study (2015), low back pain (LBP) has been one of the five leading causes of disability over the past 30 years. The indirect costs of unfulfilled work and social benefits due to LBP are several times higher than the costs of actual treatment [1,2]. In the internal structure of the LBP, nonspecific or musculoskeletal pain accounts for up to 80–90% of cases. LBP is characterized by a high prevalence, high material costs for rehabilitation, and a large polymorphism of clinical manifestations. The number of treatment and diagnostic methods with a well-founded evidence base were small. The main assessment of the effectiveness of therapy in most cases is based on the patient’s subjective feelings and data from various questionnaires and scales. The subjunctivization of therapy evaluation leads to the underestimation of many treatment methods, which are questioned from the standpoint of evidence-based medicine. With this type of back pain, during clinical examination, radiography and MRI are inappropriate because of the lack of correlation between the severity of degenerative changes in the spine and the clinical picture [3,4,5].

The presence of pain causes suffering and a reduction in the quality of life in a person, and therefore nonsteroidal analgesic drugs remain the first line drug of choice. However, they simultaneously have, especially with uncontrolled use, side effects on the gastrointestinal tract, cardiovascular and genitourinary systems, and other organs and systems [6].

Nondrug therapy is an important curative factor [7]. One of the additional methods of treatment positively proven for various pain syndromes is percutaneous (transcutaneous) electro-neurostimulation (TENS) [8,9]. The Cochrane Review (2015) showed the efficacy of TENS for acute pain of various origins. A comparative analysis with a placebo was given with recommendations to consider TENS as a method of treatment, prescribed alone or in combination with other therapies [10]. The method was also effective in treating neck pain in patients with whiplash injury after car accidents [11].

In addition to the subjective methods of assessing various methods and diagnostics of LBP treatment, passive microwave radiometry (MWR) can be chosen. The method is based on measuring the intensity of its own electromagnetic radiation of the internal tissues of the body in the ultra-high-frequency range. MWR is widely used for various pathological conditions. Processes occurring in the tissues of the body, especially those associated with pain, are often accompanied by a universal pathophysiological reaction. One of the signs of inflammation is an increase in temperature, which is associated with metabolic, vascular, and/or regulatory processes. Stable temperature changes precede or accompany clinical and morphological manifestations of the pathological process. Therefore, with a high probability, MWR can be used for early diagnosis and objective monitoring of treatment effectiveness [12,13].

## 2. Goal

In this study, we used MWR to assess the effectiveness of various therapeutic regimens in patients with acute and subacute nonspecific LBP.

## 3. Materials and Methods

The study was carried out at the Problematic Scientific Laboratory of Physical Methods of Diagnosis and Treatment of Rostov State Medical University. A clinical randomized, parallel, controlled, prospective study of patients with LBP was carried out, with patients divided into two groups (group selection method, even or odd. The local independent ethical committee of Rostov State Medical University approved the study (protocol No. 10/19 of 30 May 2019), and informed consent was obtained from all patients.

The total duration of the disease in patients ranged from 2 to 20 years, and the current exacerbation ranged from 1 day to 3 months. Clinical and neurological examination of patients with LBP was performed according to a specially developed protocol. The protocol included personal data, anamnestic data (including the duration of the current exacerbation), concomitant diseases, objective data, neurological status (including possible symptoms of radiculopathy and myelopathy), and a questionnaire for diagnosing neuropathic pain (Douleur Neuropathique en 4 questions (DN4)). The inclusion criteria included patients with acute and subacute LBP of the musculoskeletal system, with a nociceptive (with the duration of the current exacerbation up to 3 months inclusive) and nonspecific character, caused by various degenerative-dystrophic lesions of the spine or paravertebral tissues (without specifying the source of pain) who were in outpatient or inpatient departments. The exclusion criteria included presence of radiculopathy and myelopathy, known congenital anomalies of the spine, ankylosing spondylitis, reactive arthritis, rheumatoid arthritis, suspicion of a secondary nature of pain, gross cardiac arrhythmias, and probable neuropathic pain. Before treatment, some patients underwent MRI of the spine without axial load to exclude other diseases of the lumbar spine.

A 10-point visual analogue scale (VAS) was used to assess the intensity of pain syndrome [14]. The groups were composed of patients with an initial VAS score of 6–10 points.

Group I received traditional syndrome-oriented conservative treatment: ketoprofen, tolperisone, symptomatic slow-acting drugs in osteoarthritis-SYSADOA (glucosamine + chondroitin sulfate) [3,4,15] (n = 30, average age 54.9 ± 2.3 years); MRI was performed in 7 patients (Toshiba Vantage Elan 1/5 Tl, Toshiba, JapanM).

Group II received a combination of traditional treatment with percutaneous electroneurostimulation (TENS) using a Self-Controlled Energy Neuroadaptive Regulator SCENAR-CHENS-01 device (ZAO OKB RITM, Taganrog, Russia), (n = 25, age 52.8 ± 2.5 years); MRI was performed in 3 subjects (Toshiba Vantage Elan 1/5 Tl).

**Figure 1:**
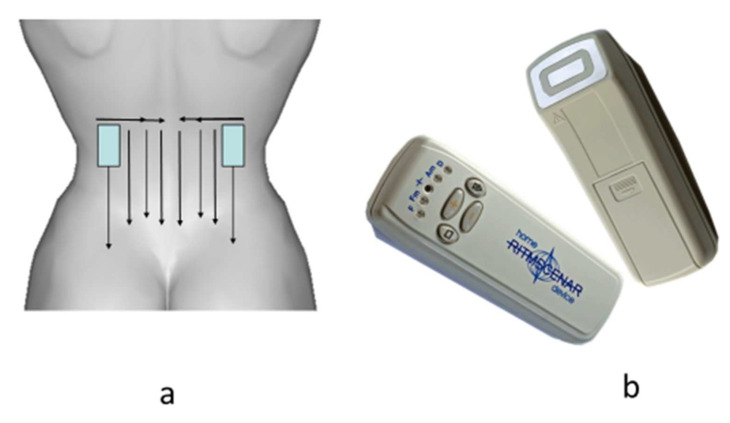
The template of application of SCENAR in the lumbar region (**a**) and type of device (**b**).

The procedures were carried out for the first 3 days every day and then every other day (only 8–10 procedures per course). The total time of the procedure was up to 20 min. The template and the type of device are shown in Figure 1.

We used a subjectively dosed regimen, which is preferable for clearly expressed locality of symptoms, in case of acute and well-localized complaints, and in the treatment of affected areas of a relatively large area. The zones of influence were determined before the start of the procedure according to the primary complaints (the area of pain indicated by the patient), and during treatment according to secondary signs. The processing time for primary complaints was 3–5 min before the clear appearance of secondary signs, the zones of small asymmetry.

**Figure 2:**
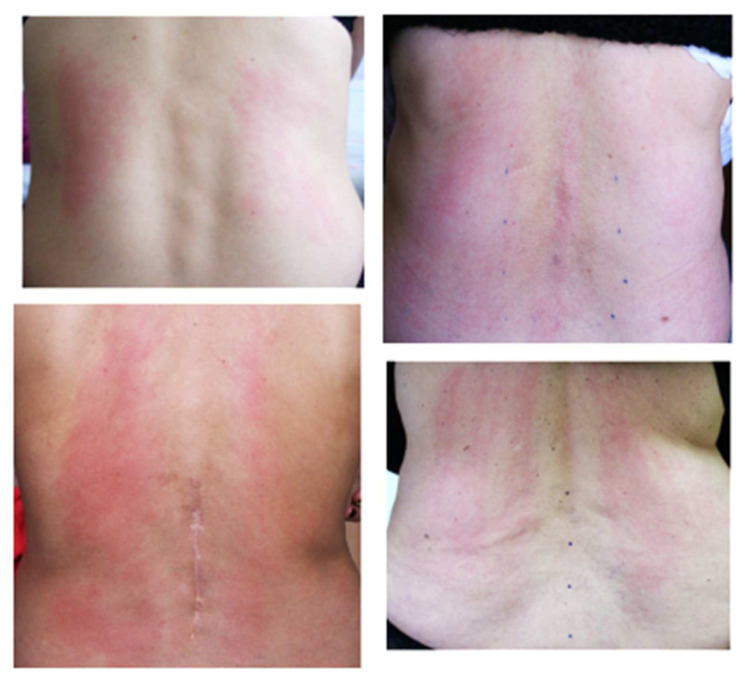
Examples of asymmetry.

Secondary signs included changes in the characteristic sound under the electrode, the effect of sticking the electrode of the apparatus to the skin when moving, depending on local sweating (the electrode was held until it was displaced from this area of the skin), changes in skin color in the treatment area, hyperthermia or blanching of certain areas, etc. (Figure 2).

During treatment, several zones appeared with qualitatively different secondary signs. In this case, the area for additional processing was determined by the intensity of manifestation of signs (the most localized hyperthermia, pronounced change in sound under the electrode, and the painful area). Usually, the secondary signs appeared in 10–15 min.

The perception of the device’s impact is largely subjective. Based on the patient’s feelings, we distinguished the following modes of exposure: comfortable (a feeling of slight tingling and vibration), intense (a feeling between the comfort and pain threshold), and weak (the patient does not feel or almost does not feel a tingling sensation).

Energy was selected outside the processing zone. In most cases, a comfortable treatment regime was used. During the procedure, the energy did not change. With a clear localization of complaints, an intensive regimen was used, which passed the level of the pain threshold. A stimulation frequency of 90 Hz was applied.

To objectively control the treatment, passive MWR was used MWR2020 (former RTM-01-RES) (Medical Microwave Radiometry Ltd., Edinburgh, UK) with an antenna width of 39 mm. We collected the MWR data of 31 healthy volunteers (mean age 54.9 ± 1.1 years). The internal (microwave emissions) and skin temperatures (infrared emissions) of the spine were measured simultaneously, in a sitting position, along the median and paravertebral (30 mm from the median) lines on the left and right, at the level of the spinous processes of the five lumbar vertebrae (five sensor positions, 15 in total). For 5–7 min before the examination, the skin of the back was opened for adaptation. The humidity in the room was in the range of 50–60%. The studies were carried out under ambient temperature in the morning from 9 to 11 a.m. The temperature in the research room in both groups at all stages of measurement corresponded to the optimal range (22.0–26.9 °C) [16].

**Figure 3:**
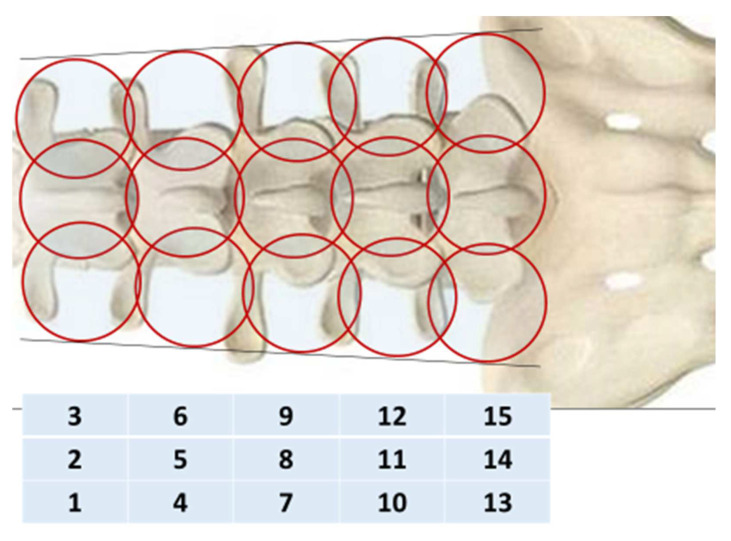
Diagram of the sequence of sensor placement in the study of the lumbar vertebrae by the MWR.

The internal (microwave) and skin (infrared) temperatures were measured (Figure 3) Thermal asymmetry was also visually determined in each patient through visualizations of both temperature fields before and after treatment. We also determined ΔT = Tmax − Tmin for internal and skin temperature in the control and in patients in 2 groups before and after treatment.

## 4. Statistical Analysis

Quantitative indicators are presented in the form of a sample mean with an error of the mean. The samples were tested for normal distribution using the Shapiro–Wilk test. When the value of *p* ≥ 0.05, the null hypothesis was accepted, that is, the sample obeyed the normal distribution law, and the parametric Student’s *t*-test was used to compare the values of indicators in the groups. When comparing before and after treatment within one group, the samples were dependent, and when comparing indicators between Group I and Group II, the sample was independent. The Mann–Whitney test was used in the absence of a normal distribution. The Wilcoxon test was used to compare the dynamics of the mean values of the dependent samples. Differences were considered statistically significant at *p* ≤ 0.05. STATISTICA (Version 12.0 StatSoft, St Tulsa OK, USA) and Microsoft Office Excel (version 2010, Microsoft, Redmond, WA, USA) were used for statistical analysis of the results.

## 5. Results

At the first questioning of the patient before treatment, the average VAS score in Groups I and II did not significantly differ and ranged from 6.73 to 6.24 points. The results of treatment based on pain assessment are presented in Table 1. During therapy in both groups, the severity of pain at each stage significantly reduced in comparison with the previous one. In Group II with SCENAR included, at all measurement points, the analgesic effect was significantly higher than in Group I, and at the end of treatment it was almost two times lower (1.28 ± 0.21 and 3.00 ± 0.25 points, respectively).

When analyzing the data, we found that the changes in skin temperature in both groups during treatment were unreliable. Individual fluctuations in the internal temperature Tmax ranged from 31.6 to 38.4 °C in our patients. The results of the changes in the average internal MWR data are presented in Table 2.

Table 2 shows that changes in depth Tmin in comparison with the control, as well as at the beginning and end of treatment, were not significant. In Groups I and II, we noted an increase in the maximum depth temperature by 0.74 and 0.83 °C, respectively, in relation to control before treatment.

In Group I, after the end of treatment, the internal Tmax decreased by 0.23 °C and remained significantly higher than that of the control by 0.51 °C. In Group II, when SCENAR was turned on, the depth Tmax normalized (decrease by 0.48 °C). Table 2 contains averaged data, which enables an assessment of the tendencies of local temperature disturbances, primarily in large groups of patients, to conduct a comparative analysis of treatment.

**Figure 4:**
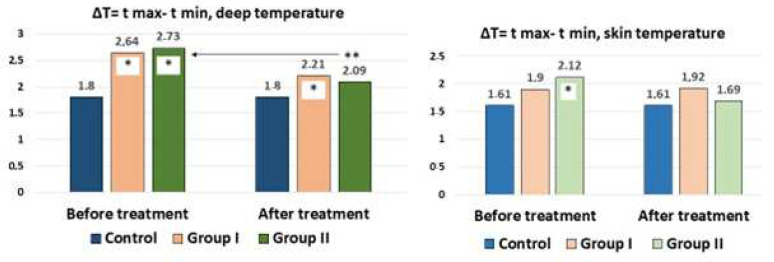
Temperature gradients of internal and skin temperatures in the lumbar region during traditional treatment (Group I) and for combination treatment with the inclusion of percutaneous neurostimulation (Group II) in comparison with the control group. * Statistically significant difference in relation to control; ** in relation to their group before treatment at *p* < 0.05 (assessment by the Mann–Whitney test).

A healthy individual or with a pathological process, a priori, will have areas with a higher or lower internal temperature. In the mosaic of the inflammatory (or proliferative) process, atrophy (or low vascularization and blood flow) provides the basis for the temperature gradient. In our case, among the 15 measurement zones in the lumbar region, areas with lower and higher temperatures were recorded. Determination of the temperature gradient, in our opinion, allows determination of the pathological changes and the result of treatment. The ΔT = Tmax − Tmin for internal and skin temperature in the control and in patients before and after treatment is shown in Figure 4.

It can be seen from the figure that the ΔT of the internal temperature (average value) was 1.80 °C for the norm and 1.61 °C for the skin temperature. With LBP, there was a significant increase in the ΔT of the internal temperature up to 2.64 °C in Group I and 2.73 °C in Group II, which was most likely associated with aseptic inflammation. When analyzing the ΔT of the skin, an increase in the gradient from 1.61 to 1.90 °C in Group I and 2.1 °C in Group II was additionally noted. With the traditional treatment of patients, a decrease in the gradient of the depth temperature to 2.21 °C was noted, which, however, was significantly higher than that of the controls. In Group II with percutaneous neurostimulation, the decrease in the depth temperature gradient did not significantly differ from that of the control. In Group I, the temperature gradient after treatment remained at the same levels, and in Group II, a decrease in the gradient in comparison with the control was noted.

The MWR method is primarily positioned as personalized diagnostics, and it is of clinical interest to study internal temperature fields in real time to identify thermal asymmetry in a particular patient.

Because each patient differed in the severity and duration of pain, the degree of quality of life disorders, as well as the localization of the pathological process, we present several clinical examples from each group.

**Figure 5:**
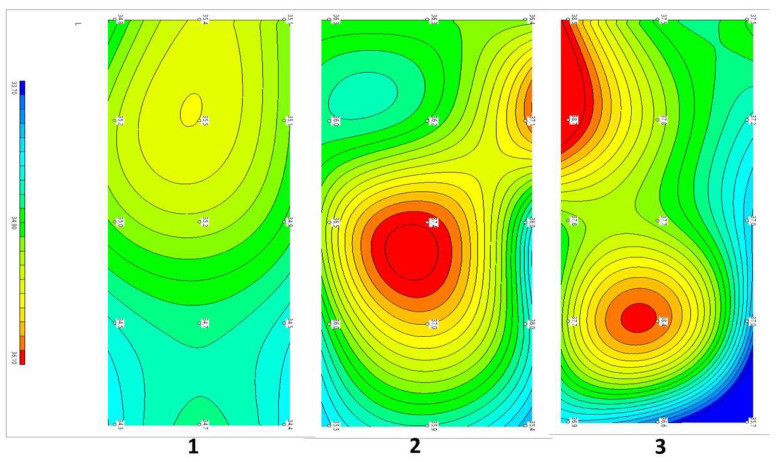
Dynamics of temperature fields in the lumbar region in patient P against the background of standard treatment. (**1**) Healthy volunteers, mean data, n = 31. (**2**) Patient P, before treatment. (**3**) Patient P, after treatment. Note: On the left, the fields of internal temperatures, from left to right indicate L1 to L5 (in the center in the projection of the spinous processes; above and below, paravertebral 30 mm from the center); on the right, thermograms at 15 measurement points.

Example 1: Patient P 58 years old. Diagnosis: Dorsopathy of the lumbosacral spine, lumbodynia, and muscle-tonic syndrome. Concomitant diagnosis: Chronic cholecystitis and remission. Recurrent low back pain for approximately 20 years. The duration of the current exacerbation was three days. The pains localized in the projection of the spinous processes L2–4, subjectively in depth. There were no neurological deficits. The tension symptoms were negative. Therapy was traditional and conservative. VAS before treatment: five points, after three weeks: two points. The dynamics of changes in temperature fields and thermograms are shown in Figure 5.

Compared with the symmetric temperature field of the control group (1) before treatment, in patient P...a (2), zones of increased temperature were visualized in the L2 projections on the right, paravertebral, and L3–4 above the spinous processes. The ΔT = Tmax − Tmin depth temperature before treatment was 2.0 °C and that of the skin was 2.7 °C. After treatment (3), the asymmetry did not disappear. There were zones of increased temperature in the L1–2 projection on the left paravertebral area, and elevated temperature in the L5 projection in the center. As we can see from this example, the decrease in pain according to the VAS from five to two points in this patient with traditional treatment did not correlate with temperature asymmetries or temperature normalization.

**Figure 6:**
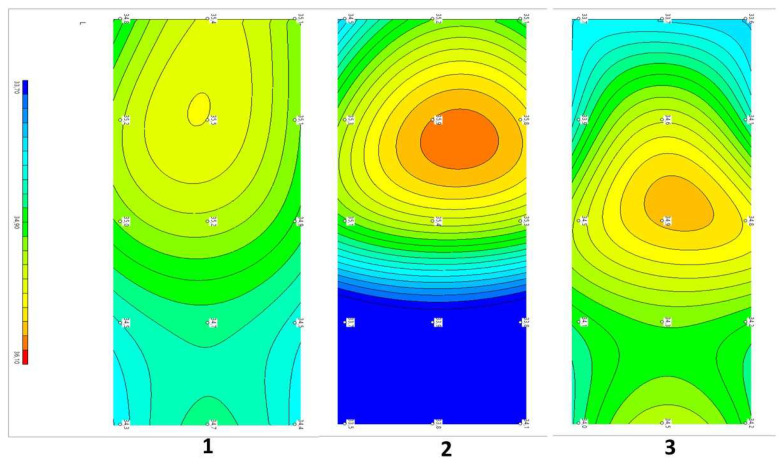
Dynamics of temperature fields in the lumbar region in patient In against the background of complex treatment. (**1**) Healthy volunteers, mean data, n = 31. (**2**) Patient Iin, before treatment. (**3**) Patient I.in, after treatment. Note: On the left, the field of internal temperatures, from left to right indicate L1 to L5 (in the center of the projection of the spinous processes; above and below, paravertebral 30 mm from the center); on the right, a thermogram of 15 measurement points.

Example 2: Patient I...in, 56 years old. Diagnosis: Dorsopathy of the lumbosacral spine, lumbodynia, and muscle-tonic syndrome. Concomitant diagnosis: Arterial hypertension in the first stage. Recurrent low back pain for more than 30 years. The duration of the current exacerbation was three days. The pain localized in the lumbar region, subjectively in depth, paravertebrally on the left in the projection L1–2. There were no neurological deficits. The symptoms of tension were negative. Complex therapy with the addition of eight sessions of SCENAR therapy VAS before treatment was six points, and after three weeks of treatment was two points. The dynamics of changes in temperature fields and thermograms are shown in Figure 6.

Compared with the symmetric temperature field of the control group (1) before treatment, patient (2) had visualized zones of increased temperature in the L2 projections above the spinous process and on the right, paravertebrally. The ΔT= Tmax − Tmin depth temperature before treatment was 2.4 °C and that of the skin was 1.3 °C. After treatment (3), the asymmetry significantly decreased. There was a zone of slightly elevated temperature in projection L3 above the spinous process. The ΔT = Tmax − Tmin internal temperature after treatment decreased and amounted to 1.0 °C, and −1.1 °C for the skin.

This example shows that the decrease in pain from six to two points, in this patient with complex treatment, correlated with a decrease in Tmax and Tmin; the asymmetry of the fields decreased, and the temperature normalized with a decrease in the ΔT of the depth and skin temperatures.

## 6. Discussion

TENS has long been used for various pain syndromes. Currently, the mechanism of the analgesic efficacy of various devices and techniques is mainly associated with the effects of neuropeptides and other regulatory peptides [10]. Unlike other applied TENS, SCENAR stimulates C-fibers, which most actively respond to electrical stimulation. Thus, SCENAR therapy can reliably reduce the severity of neck pain according to the VAS and the neck disability index (NDI) in comparison with the TENS group. SCENAR therapy is superior to TENS therapy in terms of analgesic effects and NDI for whiplash injuries [11].

Some TENS problems remain unresolved, such as the feeling of extinction of the energy of stimulation in the patient due to the development of tolerance, which leads to the need for increasing the strength of the impact. The other problem is the static treatment of certain areas of the skin with various sticky and metal electrodes, which significantly reduces the effectiveness of the treatment.

In recent decades, devices for a new generation of TENS have been developed, including SCENAR. The device has been approved for use in many countries worldwide. SCENAR properties that distinguish it from previously used TENS [17]. It is possible to carry out SCENAR examination of the skin condition by its impedance by changing the pulse characteristics and both static and dynamic impacts (movement of the device on the skin). The device has a high-amplitude, nondamaging effect achieved using a short, single, bipolar 200–300 μs pulse. The pulse is variable and changes during therapy, which depends on the electrical conductivity; therefore, the energy of the impact does not change according to the patient’s subjective sensations. Its current density is high, which is orders of magnitude higher than that of the known methods of electrotherapy. The amplitude of the first half-wave depends on the patient and varies from 10 to 100 mA.

In our patients, we not only determined the effect of potentiating analgesia; we also used MWR to assess the treatment efficiency. We found a decrease in internal temperature and asymmetry in LBP patients after treatment. We observed a decrease in the aseptic inflammatory process in the affected segment due to the SCENAR-therapy inhibition of myeloperoxidase in inflammation and activation of antiradical enzymes [18]. In a study on healthy volunteers, after the use of InterX^®^ 5002 (Neuro Resource Group Inc., Richardson, TX, USA) (an analogue of SCENAR), a more pronounced response was observed than after TENS in the cascade of proinflammatory cytokines with their decrease. The authors attributed this effect to the design features of the device owing to the higher amplitude (approximately 3–5 times higher) and current density (approximately 90 times higher) at the output of the InterX^®^ 5002 in comparison with TENS Biomed 2000XL® (BioMedical Life Systems, Carlsbad, CA, USA) [19].

MWR differs from other imaging methods. The method does not examine deviations in the anatomical structure of internal tissues or indirect signs of inflammation and destruction, but rather examines the deviations in temperature. In the case of inflammatory processes, they affect the distribution of temperatures in internal tissues and often precede structural changes.

It is safe for most people to use SCENAR, and they will not usually experience any side effects. However, the electrical impulses that SCENAR produces may cause a buzzing, tingling, or prickling sensation, which some people may find uncomfortable. Some people may be allergic to the materials. Anyone who experiences skin redness and irritation can switch to using hypoallergenic ones.

Whereas MWR is an attractive method, the device can be improved. The current diameter of the MMWR2020 antenna is too large for measurements on nonflat human body surfaces (i.e., head) or on children. The device can be scaled down and wirelessly connected.

It would be ideal to combine SCENAR and MWR into one device so that the specialist would be able to identify an abnormal spot and accordingly vary the electrical parameters.

## 7. Conclusions

An increase in the analgesic potential of therapy when using a combination treatment with SCENAR therapy, normalization of local depth temperature, assessed using MWR, allows a sufficiently reliable comparative analysis of various treatment and diagnostic methods. Any new treatment and diagnosis method requires the accumulation of clinical material. It is still premature to draw final conclusions regarding the use of MWR in lumbodynia. It is necessary to continue the collection of clinical data, both in patients and healthy people, to correlate MWR asymmetries with neuro-orthopedic and MRI data.

Our experience with using MWR indicates the potential of using this method for objectifying the current state of a particular patient with LBP, minimizing the occurrences of simulation and aggravation, monitoring the effectiveness, and objectively comparing various treatment regimens. MWR asymmetry visualization allows identification of a zone of pathological muscle spasm or other process with increased metabolism for the possible targeted use of treatments such as TENS, massage, manual therapy, etc.

## Figures and Tables

**Table 1:** Dynamics of BNS assessment by VAS (M ± m).

Groups	Days/VAS			
	1 Day	3 Day	7 Day	19–21 Day
	1	2	3	4
Group I	6.73 ± 0.27	5.60 ± 0.25 P_2–1_ *	4.33 ± 0.24 P_3–1_ *, P_3–2_ *	3.00 ± 0.25 P_4–1_ *, P_4–2_ *, P_4–3_ *
Complex therapy Group II	6.24 ± 0.28	4.64 ± 0.27 ** P_2–1_ *	3.04 ± 0.22 ** P_3–1_ *, P_3–2_ *	1.28 ± 0.21 ** P_4–1_ *, P_4–2_ *, P_4–3_ *

Note: * difference in mean value dynamics at *p* < 0.05 (Wilcoxon test); **difference between groups in pairwise comparison at *p* < 0.05 (assessment by the Mann–Whitney test).

**Table 2:** Indicators of deep temperature in the lumbar region with various methods of treatment of patients with LBP (M ± m).

Parameters	Control	Group I (Standard Therapy)		Group II (Combination Therapy)	
		before Treatment	after Treatment	before Treatment	after Treatment
MWR Internal temperature °C					
Tmin	33.72 ± 0.19	33.62 ± 0.16	33.82 ± 0.15	33.62 ± 0.28	33.78 ± 0.20
Tmax	35.52 ± 0.15	36.26 ± 0.18 *	36.03 ± 0.17 *	36.35 ± 0.26 *	35.87 ± 0.20

Indices of internal temperature in the lumbar region with various treatments with LBP (M ± m) Note: * statistically significant difference in relation to control at *p* < 0.05 (assessment by the Mann–Whitney test).
